# Efficacy of the Aussie Optimism Program: Promoting Pro-social Behavior and Preventing Suicidality in Primary School Students. A Randomised-Controlled Trial

**DOI:** 10.3389/fpsyg.2017.01392

**Published:** 2018-02-26

**Authors:** Clare M. Roberts, Robert T. Kane, Rosanna M. Rooney, Yolanda Pintabona, Natalie Baughman, Sharinaz Hassan, Donna Cross, Stephen R. Zubrick, Sven R. Silburn

**Affiliations:** ^1^School of Psychology and Speech Pathology, Curtin University of Technology, Perth, WA, Australia; ^2^Child Health Promotion Research Centre, Edith Cowan University, Perth, WA, Australia; ^3^Telethon Kids Institute, University of Western Australia, Perth, WA, Australia

**Keywords:** suicidality, anxiety, depression, primary school children, Aussie Optimism Program

## Abstract

The efficacy of an enhanced version of the Aussie Optimism Program (AOP) was investigated in a cluster randomized controlled trial. Grade 6 students aged 10–11 years of age (*N* = 2288) from 63 government primary schools in Perth, Western Australia, participated in the pre, post, and follow-up study. Schools were randomly assigned to one of three conditions: Aussie Optimism with teacher training, Aussie Optimism with teacher training plus coaching, or a usual care condition that received the regular Western Australian Health Education Curriculum. Students in the Aussie Optimism conditions received 20, 1-h lessons relating to social and interpersonal skills and optimistic thinking skills over the last 2 years of primary school. Parents in the active conditions received a parent information booklet each year, plus a self-directed program in Grade 7. Students and parents completed the Extended Strengths and Difficulties Questionnaire. Students who scored in the clinical range on the Emotional Symptoms Scale were given The Diagnostic Interview for Children and Adolescents IV, to assess suicidal ideation and behavior, and depressive and anxiety disorders. Results indicated that Aussie Optimism with teacher training plus coaching was associated with the best outcomes: a significant increase in student-reported pro-social behavior from pre-test to post-test 1 (maintained at post-test 2) and significantly lower incidence rates from suicidal ideation at post-test 2 and follow-up. No significant intervention effects on anxiety and depressive disorders, and total difficulties were reported. These findings suggest that the AOP with teacher training along with coaching may have the potential to positively impact on suicidality and pro-social behavior in the pre-adolescent years.

## Introduction

Mental health problems are now ranked as a leading cause of the burden of disease worldwide. Specifically, according to the most recent Burden of Disease Study (Australian Institute of Health and Welfare, 2016) 24% of the non-fatal burden of disease is caused by mental and substance use disorders in Australia, and almost 12% of the overall burden which includes premature death by suicide or self-inflicted injuries. To reduce this level of health burden, we need to engage in more prevention and mental health promotion at a number of levels and specifically target anxiety and depression before they emerge. In addition, anxiety and depression and depression disorders rank among the five leading causes of the total burden of disease for children and adolescents aged 5–14 years (Australian Institute of Health and Welfare, 2016). In this age group, slight gender differences are apparent, with anxiety and depression disorders making up 15.8% of the burden for males and 19.4% for females (Australian Institute of Health and Welfare, 2016). Interventions with children and adolescents hold promise because they can prevent the development of internalizing problems at key developmental time points, as well as building resilience and positive developmental trajectories at times of transition such as the move from primary to secondary school (Neil and Christensen, 2009; Durlak et al., 2011).

Reviews of prevention programs to treat internalizing disorders such as anxiety (Feldner et al., 2004; Neil and Christensen, 2009; Rapee et al., 2009) and depression (Merry et al., 2004; Horowitz and Garber, 2006; Stice et al., 2009) have supported the efficacy of both universal and targeted programs for and anxiety and depression (Ahlen et al., 2015). Targeted prevention programs are offered to groups who are at-risk of developing psychological disorders, while universal programs are delivered to the whole population, such as whole school classes regardless of risk status (Merry et al., 2011). Merry et al. (2004) found that only targeted psychological interventions achieved significant reductions in depression post-intervention, and Stice et al. (2009) reported larger effect sizes for programs targeting depression in high-risk adolescents. Rapee et al. (2009) found small but significant effects for a universal anxiety prevention program, with stronger effects for children versus adolescents; while Neil and Christensen’s (2009) review indicated efficacy for both universal and targeted school-based anxiety prevention programs; however, indicated interventions, i.e., programs that are delivered to groups or individuals who exhibit early symptoms of psychological disorders, have shown more promise (Feldner et al., 2004). These studies are based on a deficit specific model rather than a competence enhancing model. Deficit specific models aim to prevent and reduce behavioral problems via utilizing a single intervention with specific mechanisms and take less consideration of other factors. Conversely, the competence enhancing model primarily focuses on building and promoting social-emotional well-being by assimilating the social, emotional, interpersonal skills, behaviors and cognitive components (Greenberg et al., 2003).

Payton et al. (2008) reviewed 180 school-based studies that investigated SEL curricula. These programs included identification of feelings and problems, goal setting, conflict resolution, and social problem solving strategies. Small to moderate effect sizes were found for school achievement (ES = 0.28) and for school grades (ES = 0.34). Importantly, these effects were achieved with regular teaching and support staff using programs that focused on sequenced instructions, active learning strategies, and targeted social and emotional skills.

Criticisms of universal depression prevention programs suggest that the intervention dose may not be sufficient when implemented with classes of 20–30 students, that there is a lack of ability to individualize program content, and a lack of symptom change in the majority of students (Stice et al., 2009). Spence and Shortt (2007) have also commented on low power, limited follow-ups, lack of random assignment, use of no-intervention control conditions, few diagnostic measures and limited use of blind assessors. While many universal studies report non-significant results (Merry et al., 2004, 2011), there is evidence that statistical power may be a problem. Cuijpers (2003) indicates that the low base rate of depression in children and adolescents and small effect sizes for universal trials, make it difficult to detect effects without a substantial number of participants. One solution is to focus universal interventions on multiple disorders such as anxiety, depression, and suicidal behaviors.

The Aussie Optimism program (AOP; Roberts et al., 2010) is a universal mental health promotion strategy designed to reduce and prevent anxiety, depression, and suicidal behavior in young adolescents. The current study involved evaluating two versions of the AOP, each containing two 10-week school-based components—Social Life Skills (SLS; Roberts C. et al., 2003) and Optimistic Thinking Skills (OTS; Roberts R. et al., 2003) implemented in Grades 6 and 7, respectively, plus a self-directed program for parents and families (PF; Drake-Brockman and Roberts, 2001) to be implemented in the second half of the last year of primary school. The difference between the two versions is that one version involved coaching in addition to the standard training to help teachers implement the program with fidelity, and to manage implementation issues with schools.

The content of the AOP: SLS and OTS programs target social, emotional, and cognitive skills. Following are the key components of the SLS and OTS programs. *SLS*: (1) identifying feelings, (2) decision making, (3) communication, (4) assertiveness and negotiation, (5) coping skills, and (6) social support networks. *OTS*: (1) connecting thoughts and feelings, (2) thinking styles, (3) challenging negative thinking styles, and (4) preparing for adolescence.

Intervention in late childhood provides an opportunity to act before prevalence rates of these disorders increase in adolescence in conjunction with the enhanced cognitive abilities that may result in pessimistic attributions, negative beliefs, and more obvious peer perceptions of social incompetence (Seroczynski et al., 1997). Teaching social skills, problem solving, and cognitive attribution training, provides students with effective resilience skills. Enhancing protective factors, such as social support is also beneficial. The PF program (Drake-Brockman and Roberts, 2001) is provided for parents to enhance family protective factors, around the transition to high school. The program is implemented in Grade 7, the final year of primary school. The content is based on theories of depression, anxiety and suicide prevention, and incorporates validated techniques to change emotions, cognitions and behavior (e.g., Stark, 1990; Seligman et al., 1995; Kendall, 2006; Payton et al., 2008). The key components of the PF program are: (1) preparing for challenges ahead, (2) working together as a family, (3) optimistic thinking, (4) friendship, peer pressure, and bullying, and (5) preparing for adolescence.

Earlier versions of the AOP implemented in Grade 7 have resulted in fewer depressive symptoms and more positive self-worth amongst adolescent girls 6 months after moving to high school (Quayle et al., 2001). In a randomized controlled trial, conducted with disadvantaged schools, parents reported significantly fewer internalizing problems immediately after the intervention (Roberts et al., 2010). The above mentioned studies provide initial evidence that the combination of the SLS, OTS, and PF components has the potential to reduce anxiety, depression, and suicidality through building resilience and social-emotional and cognitive skills in children and pre-adolescents.

Studies investigating the efficacy of other AOP have shown effective intervention effects in children aged 8 and 9 years old. The Aussie Optimism Program: Positive Thinking Skills (AOPTS) was designed to prevent anxiety and depression in Grade 4 and 5 students. A pilot study of AOPTS indicated that children who received the program had fewer depressive symptoms and disorders at post-test and 9-month follow-up (Rooney et al., 2006). A larger randomized controlled trial with 910 students was conducted and found that, compared to the control group children, intervention children were significantly less depressed at post-test and had lower levels of parent-reported total difficulties immediately after the intervention; these effects were maintained at the 6-month follow-up (Rooney et al., 2013a). The same cohort of children was followed up at 30 months and reported significant reductions in hyperactive behaviors (Rooney et al., 2013b).

The purpose of the current study was to evaluate the efficacy of two delivery strategies of the AOP: SLS and OTS programs in combination with the self-directed AOP for PF. The study employed two active implementation strategies and one usual care control condition, the regular Western Australian Health Education Curriculum (Curriculum Council Western Australia, 2001). The teacher training condition involved 16 h of teacher training in the implementation of the programs. The second strategy included the same 16 h of teacher training with the addition of 10, 1-h coaching sessions for teachers over the 2 years of program implementation. The coaching sessions were designed to support teachers with program implementation and help principals and teachers to individualize and institutionalize the program. Outcomes for both a universal sample including all children regardless of their symptom levels, and an indicated sample of children who were already experiencing clinical levels of emotional symptoms, were measured. First, it was hypothesized that both active intervention conditions would show better mental health outcomes and more pro-social behaviors, compared to the usual care control group. Second, it was hypothesized that the indicated sample from both intervention groups would show reduced incidences of anxiety and depressive disorders and suicidal ideation/behaviors, and increased recovery from these clinical disorders compared to the usual care control group. We expected to see these effects maintained at both post-test and 1-year follow-up.

## Materials and Methods

### Participants

Sixty-three (69.2%) of the 91 government primary schools from three education districts in Western Australia (Fremantle, Rockingham, and Mandurah) were recruited to the study. Of the 3288 Grade 6 students enrolled in the recruited schools, 69.9% of the students (*n* = 2288) and 63.8% (*n* = 2097) of their parents agreed to participate. The students were aged between 9.67 and 12.45 years (*M* = 11.05, *SD* = 0.33); 48.9% (*n* = 1118) were female and 51.1% (*n* = 1170) were male. There were 863 students in the training only group, 794 students in the training/coaching group, and 630 students in the usual care control group.

There were no significant between-group differences in student age, *F*(2,2282) = 0.793, *p* = 0.466, and male/female ratio, χ^2^ (2, *n* = 2287) = 1.26, *p* = 0.532. There were no significant between-group differences in school socio-economic status (SES), *F*(2,60) = 0.15, *p* = 0.86, school size, *F*(2,60) = 0.76, *p* = 0.47, or the number of Grade 6 students in the schools, *F*(2,60) = 0.52, *p* = 0.60. Of the 2076 students who reported their ethnic origin, 80.7% (*n* = 1675) identified as Australian, 1.7% (*n* = 36) as Australian Aboriginal, 9.2% (*n* = 191) from other English speaking countries, 5% (*n* = 104) as Asian, 1.9% (*n* = 39) as European, and 1.5% (*n* = 31) from other non-English speaking countries. There were no significant between-group differences in ethnic origin χ^2^ (10, *n* = 2076) = 2.51, *p* = 0.113.

A subsample of 211 students (9.2%; 76 males and 135 females) who reported abnormal pre-test scores greater than six on the Emotional Symptoms scale of the student Strengths and Difficulties Questionnaire (SDQ-S) were assessed for clinical diagnoses including anxiety, depression and suicidal ideation (see **Table 1**). This indicated group was aged between 10.49 and 12.44 years (*M* = 11.01, *SD* = 0.33). Their mean SDQ-S total difficulty score (TDS) was 19.81 (range 9–33), and their pro-social scale mean was 7.93 (range 3–10).

**Table 1:** Means and standard deviations for the subsample students (*N* = 211).

	*M*	*SD*
**Total difficulty score**		
Training	19.59	4.98
Training + coaching	19.92	4.66
Control	20.04	4.31
**Pro-social score**		
Training	8.05	1.68
Training + coaching	7.83	1.88
Control	7.76	1.55

### Research Design

The 63 schools were stratified by SES^1^, school size, and the number of Grade 6 students, and randomly allocated to training only, training/coaching, and a usual care control condition such that there were 20, 22, and 21 schools in each condition, respectively. Within schools, consenting students were assessed on four occasions: pre-test (at the beginning of Grade 6), post-test 1 (at the end of Grade 6), post-test 2 (at the end of Grade 7), and follow-up (at the end of Grade 8). The research design therefore included three categorical random effects (school, teacher, student), one categorical fixed effect (group: training only, training/coaching, usual care control), and one ordinal fixed effect (time: pre-test, post-test 1, post-test 2, follow-up).

### Measures

#### Student-Reported Primary Outcomes

The student version of the Strengths and Difficulties Questionnaire (SDQ-S; Goodman, 2001) assessed child-reported mental health problems. It consists of four, 5-item subscales for hyperactivity, emotional symptoms (anxiety and depression), conduct problems, and peer problems; subscale scores are summed to produce a Total Difficulties Scale. The SDQ-S also has a 5-item pro-social behavior subscale, which does not contribute to the TDS. The present study focused on the TDS and the pro-social score. The TDS of the SDQ correlates with the Children’s Depression Inventory 0.73 and correlates with the Revised Children’s Manifest Anxiety Scale 0.72. The emotional symptoms subscale correlates 0.67 (CDI) and 0.73 (RCMAS). The emotional problems items are: often complains of headaches, many worries, often unhappy and downhearted, nervous or clingy in new situations, many fears and easily scared.

A Cronbach’s alpha coefficient of 0.79 on the TDS for the current sample indicated good internal consistency reliability. The subscales, however, were less reliable with Cronbach’s alphas of 0.58 for peer problems and 0.69 for emotional symptoms. For the TDS, Mellor (2005) reported a 2-week test–retest reliability of 0.75 for Australian children aged 11 years and older on the TDS and cross-informant correlations of 0.43 with parents and 0.35 with teachers. The subscale scores correlate significantly with other tests such as the Child Behavior Checklist (Goodman and Scott, 1999). A cut-off score of 7 or above on the Emotional Problems subscale was used to identify an *at risk* group that would be assessed for incidence of, and recovery from, depressive, anxiety, and suicidal disorders (Goodman, 1999).

#### Clinical Diagnoses

The Diagnostic Interview for Children and Adolescents IV (DICA-IV; Reich et al., 1997), a computerized diagnostic interview suitable for children 6–16 years of age, was used to assess both previous and current depressive and anxiety disorders. The DICA-IV takes between 30 and 90 min to complete. The interviews were conducted by psychologists trained for 8 h in the use of the computerized schedule. Interview questions were presented on a computer screen and children endorsed an item by clicking “yes” or “no,” or typing a short answer. Items were read to the child and the child was given assistance to type answers if required. Students answered items assessing Major Depressive Disorder, Dysthymia, Generalized Anxiety Disorder, Specific Phobias, Panic Disorder and Obsessive-Compulsive Disorder, and items relating to suicidal ideation or behavior. Endorsement of one or more of the 10 items relating to suicidal ideation or behavior was counted as a positive case.

Reich (2000) reported that studies have found high agreement between the DICA and other measures such as the Child Behavior Checklist. Welner et al. (1987) found 81% agreement between clinical interviews and DICA-IV diagnoses. Inter-rater reliability varies across anxiety and depressive diagnoses (Klein et al., 2005), but has been reported at 0.9 for MDD (Hodges, 1990). Test–retest reliability coefficients between 0.11 and 0.50 have been reported for the DICA-IV (Reich et al., 1995).

#### Parent-Reported Primary Outcomes

The parent version of the Strengths and Difficulties Questionnaire (SDQ-P; Goodman, 1999) is designed for parents/caregivers of children aged 4–16 years. It has the same format as the SDQ-S and assesses the same child mental health problems. The present study focused on the TDS and the pro-social score. A Cronbach’s alpha coefficient of 0.85 on the TDS for the current sample indicated good internal consistency reliability. Once again, the pro-social subscale was less reliable with a Cronbach’s alpha of 0.69. For the TDS, a 2-week test–retest reliability of 0.96 and strong correlations with the Rutter Parent Scale and the Child Behavior Checklist have been reported (Goodman and Scott, 1999).

#### Program Implementation Measures

Implementation of the SLS and OTS programs was measured by teacher log books, which recorded the number of implemented activities in each of the 20 modules. The log books were completed by teachers on a weekly basis. Each implemented activity was rated 0 (not completed), 1 (partially completed), and 2 (completed in full). Ratings were summed for each module and converted to a score out of 10. In addition, five student workbooks were randomly selected from each class to corroborate program implementation. These were coded using the same metric as before.

Teachers in the coaching condition were able to access five 1-h coaching sessions per year. Coaching dealt with program content, implementation of activities, class and student issues, plus parent and ethical issues that arose during the implementation phase. It was expected that teachers who had access to additional coaching support would be better able to individualize the implementation of the programs to meet the needs of the students. The coaching protocol included asking teachers what issues they wanted to discuss, supporting and praising their efforts with implementation and other issues, providing corrective feedback as required, and reviewing the teacher logbook for program implementation and process issues. In addition, coaches checked for any problems brought up by students or parents as a result of the AOP lessons.

### Procedure

Institutional Review Board approval was granted by the Curtin University Human Ethics Committee. Principals from 91 government primary schools in the Western Australian towns of Fremantle, Rockingham, and Mandurah were invited by letter and a phone call to participate in the study. A presentation was then made to the principals and the Grade 6 and 7 teachers, following which the Grade 6 and 7 teachers were provided with information and consent forms. Teachers and principals from 63 schools agreed to participate in the study.

The Grade 6 students from the consenting schools took home information and active consent forms for their parents, with reminders approximately 2 weeks later. Student questionnaires for the pre-test (at the beginning of Grade 6), post-test 1 (at the end of Grade 6), and post-test 2 (at the end of Grade 7) were administered in class time by trained research assistants blind to group allocation. At the follow-up (at the end of Grade 8), the majority of students completed questionnaires in small groups at school. Students who could not be accessed at school received their questionnaires in the mail and returned them in a pre-paid envelope or completed them over the telephone. Parent questionnaires were either sent home with students or mailed to the parents. Parents and students who did not complete questionnaires were followed up by mail and a phone call.

Students who scored in the clinical range on the SDQ-S Emotional Symptoms subscale were interviewed with the computerized DICA. Diagnostic interviews were carried out at pre-test, post-test 2 at the end of Grade 7, and the follow-up at the end of Grade 8. Students were interviewed at their school, or by phone at the Grade 8 follow-up. Students were interviewed away from their class to avoid stigmatization. Where clinical disorders were identified, parents were contacted immediately by a psychologist and the results of the assessment were discussed with them. Parents were given general advice on how to monitor and support their children, and referrals for psychological assistance were made if parents requested this. Students with a clinical disorder at any assessment point were followed up with the DICA at all subsequent assessments.

#### Intervention Conditions

Three AOPs were used in this study: SLS (Roberts C. et al., 2003) and OTS (Roberts R. et al., 2003), both curriculum-based; and the AOP for PF, a self-directed family-based program (Drake-Brockman and Roberts, 2001). The suite of programs was implemented over 2 years: SLS in Grade 6, OTS in Grade 7, and PF in the second half of Grade 7.

SLS was developed to overcome interpersonal risks such as poor social skills and social problem solving, lack of social support (Rudolph et al., 1994), and friendship difficulties (Shanahan et al., 2008). OTS targets cognitive vulnerabilities such as pessimistic attribution style (Seligman et al., 1995), and negative self-perceptions and future expectations (Beck et al., 1979; Gibb and Coles, 2005).

The SLS and OTS programs each contain 10 60-min modules that can be incorporated into regular primary school classes for health education or personal development (see **Table 2**). The modules include didactic information, interactive activities, games, co-operative learning tasks, Health and Physical Education cross-curriculum links, worksheets, and homework activities to help students generalize skills outside of the school setting. Modules are designed for implementation by teachers with whole classes. While the standard curriculum for Health and Physical Education provides students with skills and knowledge in various aspects of health and physical education, the AOP directly targets risk and protective factors that are associated with mental health difficulties, particularly anxiety and depression. The programs aim to develop emotional, social and cognitive skills and strategies such as communication, decision-making, social awareness, self-management, coping and optimistic thinking, as competencies in these areas are associated with better mental health (e.g., Collaborative for Academic, Social and Emotional Learning, 2007). CBT strategies are combined with role plays and discussions to train students to use the new skills and strategies in and out of the classroom.

**Table 2:** Content of the Aussie Optimism Program modules.

Social Life Skills	Optimistic Thinking Skills	Aussie Optimism Program for families and parents
1. Introduction and feelings	1. Identification of feelings	1. Dealing with transitions
2. Decision making	2. Identification of thoughts	2. Working together as a family
3. Communication skills	3. Linking thoughts and feelings	3. Optimistic thinking
4. Assertiveness I	4. Different thinking styles	4. Friends
5. Assertiveness II	5. Review and quiz	5. Preparing for high school
6. Negotiation	6. Generating alternative thoughts	
7. Coping skills	7. Looking for evidence	
8. Networks	8. Challenging unhelpful thoughts	
9. Friends and family	9. Decatastrophizing	
10. Transition and review	10. Review and action plans	

Each program includes a Student and Parent booklet to accompany the SLS and OTS content taught at school. The Student booklet consists of 10 modules and is structured with the following features to enhance the program implementation, i.e., (1) Resource sheets for some of the skill-based modules, (2) A practice exercise to generalize the skills at home and community settings beyond the classroom, (3) A key message and important points from each module, (4) A rating sheet to describe enjoyment and usefulness of the module, and (5) A skills checklist for students to reflect on and assess their understanding and skills learnt. In addition, the Parent booklet was used to inform parents of the program content and provide advice on how to support their child’s use of the skills in the home environment. The Parent booklet contains 10 modules and each module provides brief information about the AOP lessons taught at school. There are no explicit instructions given to the parents on when and how to use the Parent booklet at home, however, it is expected that parents would review the Parent booklet, discuss the content and practice the skills with their child once their child had completed the lesson at school.

The self-directed AOP for PF was provided for parents of Grade 7 students in the second half of the year when schools and families were preparing the students for secondary school. It aims to enhance family protective factors for young adolescents over the transition to secondary school. Topics such as working together as a family, building new friendships, stress management in times of transition, and cultivating an optimistic perspective are discussed and families are encouraged to make plans to assist their teenagers over this time. All learning outcomes were compatible with the Western Australian Department of Education’s Curriculum Framework for Health Education.

Intervention group teachers received 8 h of training per program. For SLS and OTS this included skills practice and feedback on the program activities, and discussion of implementation issues for individual classrooms. The majority of teachers taught either SLS or OTS; teachers who taught OTS also administered the PF program in Grade 7. While teachers in both intervention groups received program manuals, resources, and student workbooks, teachers in the training/coaching condition were able to additionally access up to 5 h of coaching per year to support them in program implementation. The coaching was provided by school psychologists who were accredited trainers in AOP and had experience in school-based intervention programs. Teachers in this condition accessed coaching at their own convenience for a variety of issues including: individualizing the program to meet the needs of their students, advice on how to implement certain activities and motivate students, advice on how to adapt the content for children with special needs, encouraging parent participation, and assistance with dealing with referrals for children with more serious problems.

#### Control Group

Students in the control condition received their regular health education lessons, which were related to the development of self-management and interpersonal skills. Teachers used a variety of resources and teaching strategies.

These lessons had similar learning outcomes to AOP. Control group teachers received training and resources in AOP in the second year of the research project.

### Data Analysis

The research design generated a hierarchical data structure in which time was nested within student, student was nested within teacher, and teacher was nested within school. The psychometric data (SDQ-S total difficulties, SDQ-S social skills SDQ-P total difficulties, SDQ-P social skills) were analyzed with a Generalized Linear Mixed Model (GLMM; Bryk and Raudenbush, 1987) as implemented through SPSS’s (Version 22) GENLINMIXED procedure. In order to optimize the likelihood of convergence, a separate GLMM was tested for each outcome. Each GLMM assumed a normal probability distribution for the outcome and linked it to the fixed effects (group, time, group × time) with an identity function. If the outcome did not have a normal distribution, then the parameter estimates of the covariance matrix were computed with robust statistics.

DICA assessments were conducted at pre-test, post-test 2, and follow-up. The DICA data (incidence: yes, no; recovery: yes, no) were analyzed with a GLMM that assumed a binomial probability distribution for the outcome and linked it to the fixed effects (group, time, group × time) with a logit function. Once again, in order to optimize the likelihood of convergence, a separate GLMM analysis was run for each outcome.

Unlike repeated measures ANOVA, GLMM does not rely on participants providing data at every assessment point; GLMM uses all the data present at each assessment point thereby reducing the impact of subject attrition on statistical power. Moreover, GLMM is robust to unequal group sizes, can deal with unequally spaced data collection points, does not require equal variances at each time point or equal covariances between all pairs of time points, and is able to account for correlations among repeated measurements.

## Results

### Attrition

**FIGURE 1:**
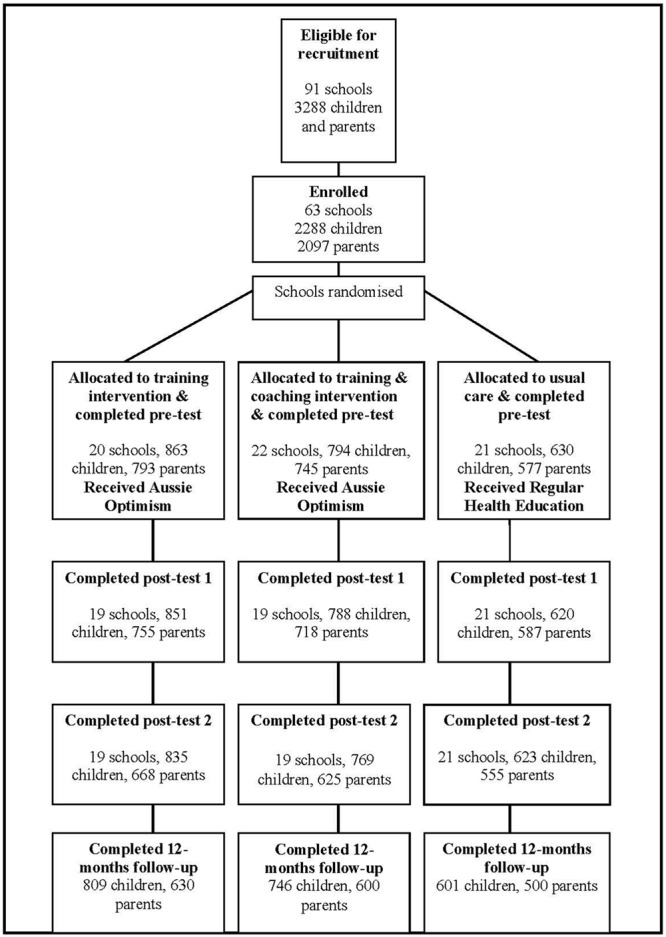
CONSORT diagram of students’ progress through the phases of the randomized control trail.

The student cohort comprised 2288 students at pre-test (the beginning of Grade 6), of which 2259 responded at post-test 1 at the end of Grade 6 (1.22% attrition), 2227 at post-test 2 at the end of Grade 7 (2.62% attrition), and 2156 at the follow-up at the end of Grade 8 (5.73% attrition rate). At post-test 1, post-test 2, and follow-up, there were no significant differences on the pre-test outcomes between those students who were retained and those who dropped out. Attrition rates did not differ significantly between the intervention and control groups. **Figure 1** provides a CONSORT (Consolidated Standards of Reporting Trials) diagram of student progress through the phases of the randomized controlled trial. The pre-test parent sample comprised 2115 parents, of which 2060 responded at post-test 1 (2.60% attrition), 1848 responded at post-test 2 (12.62% attrition), and 1730 responded at follow-up (18.20% attrition rate).

### Implementation

In 2003, 61 teachers in the training only condition implemented an average of 9.16 SLS modules (*SD* = 2.02) and 54 teachers in the training/coaching condition implemented an average of 9.24 modules (*SD* = 1.74). In 2004, 52 teachers in the training only condition implemented an average of 7.92 OTS modules (*SD* = 3.25) and 48 teachers in the training/coaching condition implemented an average of 8.06 modules (*SD* = 3.56). There were no significant between-group differences in implementation dose for SLS [*t*(113) = 0.22, *p* = 0.83] or OTS [*t*(98) = 0.21, *p* = 0.84].

In 2003, SLS coaching was offered to the 54 teachers in the training/coaching condition; and in 2004, OTS coaching was offered to the 48 teachers who remained in the training/coaching condition. Coaching was not fully utilized by teachers. The Grade 6 mean per teacher was 2.27 h (range of 0–4 h). In Grade 7, the mean per teacher was just 0.30 h (range 0–1 h). The coaching involved discussion of positive and negative aspects of the program, implementation of specific content, program structuring and timetabling, student and class issues, and teacher and parent issues. It is not clear why teachers did not fully take up the opportunity to enhance their implementation with additional coaching. Explanations included: a lack of teacher time to access coaching in their busy school days, a lack of time for school psychologists to coach teachers, teachers finding the program relatively easily to implement without coaching, and teachers not allocating time for coaching.

### Primary Analyses: Student-Reported Outcomes

#### SDQ-S Total Difficulties

The group × time interaction was significant [*F*(6,8917) = 2.35, *p* = 0.028]. LSD *post hoc* comparisons tested for changes in total difficulties between adjacent time points within each of the three groups. The comparisons indicated that the training/coaching and control groups showed a significant decrease in total difficulties from pre-test to post-test 1 (*p* < 0.001), whereas the training only group showed no change in total difficulties during this time (*p* = 0.731). Each of the three groups showed a significant decrease in total difficulties from post-test 1 to post-test 2 (all *p*s < 0.001), but there was no significant change between post-test 2 and follow-up for the training only group (*p* = 0.130), the training/coaching group (*p* = 0.107), or the control group (*p* = 0.231).

#### SDQ-S Pro-social

The group × time interaction was significant [*F*(6,8917) = 3.13, *p* = 0.005]. LSD *post hoc* comparisons tested for changes in pro-social behavior between adjacent time points within each of the three groups. From pre-test to post-test 1, there was a significant increase in pro-social behavior for the training/coaching group (*p* = 0.045), a significant decrease for the training only group (*p* = 0.009), and no change for the control group (*p* = 0.105). No changes in pro-social behavior were observed between post-test 1 and post-test 2 for the training/coaching group (*p* = 0.985), the training only group (*p* = 0.527), or the control group (*p* = 0.479). Significant decreases in pro-social behavior were observed between post-test 2 and follow-up for the training/coaching group (*p* < 0.001), the training only group (*p* < 0.001), and the control group (*p* < 0.001). Between post-test and follow-up, the time × group interaction was non-significant [*F*(2,4377) = 1.45, *p* = 0.234] indicating that pro-social behavior significantly decreased at the same rate in all three groups during this time.

#### At Risk Analysis

A binary “at risk” variable was created by assigning a “1” (at risk) to students who scored 7 or above on the Emotional Problems subscale and a “0” to students who scored below 7 (not at risk). A significant group × time interaction indicates an intervention effect. The at risk × group × time interaction was not significant for either of the student-reported outcomes. This indicates that the “at risk” factor did not moderate the previously reported intervention effects. The previously reported intervention effects therefore apply to both the “at risk” and the “not at risk” subsamples.

### Secondary Analyses: Parent-Reported Outcomes

#### SDQ-P Total Difficulties

The group × time interaction was not significant [*F*(6,7741) = 1.27, *p* = 0.270]. The group main effect was also not significant [*F*(2,7741) = 0.55, *p* = 0.574] indicating that the three groups were equivalent on parent-reported total difficulties at each of the four assessments. The main effect for time was significant [*F*(3,7741) = 40.62, *p* < 0.001] indicating that parent-reported total difficulties changed in the same manner for all three groups. LSD *post hoc* contrasts indicated that parent-reported total difficulties decreased significantly for all groups from pre-test to post-test 1 (*p* < 0.001), and from post-test 1 to post-test 2 (*p* < 0.001). In contrast, however, parent-reported total difficulties increased significantly for all groups from post-test 2 to follow-up (*p* < 0.001).

#### SDQ-P Pro-social

The group × time interaction was not significant [*F*(6,7743) = 0.44, *p* = 0.855]. The group main effect was also not significant [*F*(2,7743) = 1.71, *p* = 0.180] indicating that the three groups were equivalent on parent-reported pro-social skills at each of the four assessments. The main effect for time was significant [*F*(3,7743) = 39.43, *p* < 0.001] indicating that parent-reported pro-social skills changed in the same manner for all three groups. LSD *post hoc* contrasts indicated that parent-reported pro-social skills increased significantly for all groups from pre-test to post-test 1 (*p* < 0.001), and from post-test 1 to post-test 2 (*p* = 0.002). In contrast, however, parent-reported pro-social skills decreased significantly for all groups from post-test 2 to follow-up (*p* < 0.001).

**Table 3** provides the means and standard deviations for both the student-reported and parent-reported outcomes, broken down by group and time.

**Table 3:** Adjusted means and standard deviations for the student-reported and parent-reported Strengths and Difficulties Questionnaire.

	Pre-test		Post-test 1		Post-test 2		Follow-up	
	*M*	*SD*	*M*	*SD*	*M*	*SD*	*M*	*SD*
**Student report**					
**Total difficulty score**					
Training	10.94	6.09	10.87	6.14	9.74	5.81	9.54	5.71
Training + coaching	10.95	5.62	10.40	5.90	9.10	5.42	8.73	5.25
Control	11.09	5.79	10.49	5.86	9.70	5.63	9.43	5.30
**Pro-social score**					
Training	8.01	1.68	7.78	1.79	7.82	1.64	7.08	1.89
Training + coaching	7.59	1.76	7.72	1.77	7.72	1.75	7.16	1.84
Control	7.81	1.71	7.72	1.72	7.65	1.76	6.94	1.92
**Parent report**					
**Total difficulty score**					
Training	9.16	5.95	8.70	5.92	7.91	5.99	8.42	6.02
Training + coaching	9.24	5.95	8.77	6.11	7.90	5.75	8.44	5.77
Control	9.37	6.29	9.05	6.30	7.90	6.30	8.78	6.13
**Pro-social score**					
Training	8.03	1.81	8.22	1.74	8.33	1.75	8.00	1.85
Training + coaching	7.91	1.99	8.06	1.85	8.24	1.81	7.87	1.94
Control	7.90	1.78	8.10	1.81	8.13	1.77	7.84	1.88

### Diagnostic Outcomes

Incidence (yes, no) and recovery (yes, no) were treated as binary outcomes. Incidence was coded “0” for students who were healthy at pre-test and remained healthy at a subsequent assessment, and “1” for students who were healthy at pre-test but became clinical at a subsequent assessment. Recovery was coded “0” for students who were clinical at pre-test and remained clinical at a subsequent assessment, and “1” for students who were clinical at pre-test but became healthy at a subsequent assessment. Incidence and recovery were assessed at post-test 2 and follow-up in relation to anxiety (at least one anxiety disorder present) and/or depression (MDD or DD), and suicidal ideation. Incidence and recovery rates are reported by group and time in **Tables 4, 5**. The results of the GLMMs are reported below.

**Table 4:** Incidence of clinical disorders at post-test 2 and follow-up: proportions of training, training and coaching, and control students who changed their pre-test diagnosis from healthy to clinical.

	Pre-test	Post-test 2	Follow-up
	Healthy	Pre-test healthy to clinical	Pre-test healthy to clinical
**Depression and/or anxiety**			
Training	38	18.4% (7/38)	5.4% (2/37)
Training + coaching	34	14.7% (5/34)	3.1% (1/32)
Control	35	22.9% (8/35)	2.9% (1/35)
**Suicide risk**			
Training	40	27.5% (11/40)	2.6% (1/39)
Training + coaching	33	9.1% (3/33)	3.3% (1/30)
Control	31	22.5% (7/31)	19.3% (6/31)

**Table 5:** Recovery at post-test 2 and follow-up: proportions of training, training and coaching, and control students who changed their pre-test diagnosis from clinical to healthy.

	Pre-test	Post-test 2	Follow-up
	Clinical	Pre-test clinical to healthy	Pre-test clinical to healthy
**Depression and/or anxiety**			
Training	50	58.0% (29/50)	81.6% (40/49)
Training + coaching	24	62.5% (15/24)	84.2% (16/19)
Control	10	40.0% (4/10)	50.0% (5/10)
**Suicide risk**			
Training	48	43.8% (21/48)	74.5% (35/47)
Training + coaching	25	76.0% (19/25)	95.2% (20/21)
Control	14	64.3% (9/14)	85.7% (12/14)

#### Incidence

For depression and/or anxiety, there was no group × time interaction [*F*(1,205) = 0.30, *p* = 0.745] or group main effect [*F*(1,205) = 0.14, *p* = 0.873]. There was, however, a significant main effect for time [*F*(1,205) = 12.95, *p* < 0.001]. These results indicate that the three groups had similar incidence rates for depression and/or anxiety at post-test 2, similar incidence rates at follow-up, and showed a similar decrease in incidence rates between post-test 2 and follow-up. For suicidal ideation, there was no significant group × time interaction [*F*(2,198) = 2.84, *p* = 0.061]. There were, however, significant main effects for group [*F*(2,198) = 3.41, *p* = 0.035] and time [*F*(1,198) = 6.14, *p* = 0.014]. The main effect for group indicated that, at both post-test 2 and follow-up, the control group had significantly higher incidence rates than the training/coaching group (*p* = 0.017). No other between-group comparisons were significant. The main effect for time indicated that the three groups showed similar significant decreases in incidence rates between post-test 2 and follow-up.

#### Recovery

For depression and/or anxiety, there was no significant group × time interaction [*F*(2,156) = 0.32, *p* = 0.723] or main effect for group [*F*(2,156) = 1.99, *p* = 0.140]. There was, however, a significant main effect for time [*F*(1,156) = 5.91, *p* = 0.016]. These results indicate that the three groups had similar recovery rates for depression and/or anxiety at post-test 2, similar recovery rates at follow-up, and showed a similar increase in recovery rates between post-test 2 and follow-up. For suicidal ideation, there was no significant group × time interaction [*F*(2,163) = 0.10, *p* = 0.905]. There were, however, significant main effects for group [*F*(2,163) = 4.58, *p* = 0.012] and time [*F*(1,163) = 11.23, *p* = 0.001]. The main effect for time reflected significant post-test 2 to follow-up increases in recovery rates for all three groups. The group main effect reflected significantly greater recovery rates at post-test 2 and follow-up for the training/coaching group compared to the training only group (*p* = 0.001). The training/coaching group had higher recovery rates than the control group at post-test 2 and follow-up, but the differences were not significant. In contrast, the training only group had lower recovery rates than the control group at post-test 2 and follow-up, but once again the differences were not significant.

## Discussion

The current study found two mental health effects relating to the beneficial outcomes of the Aussie Optimism Program. Compared to students in the training only and the control group, students in the training and coaching group displayed significantly greater increases in pro-social behaviors between pre-test and post-test 1 (end of Grade 6), which were maintained at post-test 2 (end of Grade 7), and significantly lower incidence rates of suicidal ideation at post-test 2 and follow-up. Contrary to the predictions, results indicated no significant reduction in the incidence of depressive and anxiety disorders, and total difficulties at the post-test and follow-up for both intervention groups, the training only as well as the training and coaching groups.

These findings indicate that the SLS component when implemented with training and coaching was beneficial for the development of pro-social behavior for up to 1 year following the intervention. In contrast, training without coaching had a negative impact on pro-social behavior at post-test 1, suggesting that coaching should be included when implementing SLS. The fact that the pro-social behavior effects were found after the SLS component of the program had been delivered, but before OTS or the parent component of the program, suggests that the Social Skills component had successfully targeted social skills although this needs to be investigated further in future trials by examining the mechanisms by which change occurs.

The finding that the training only condition was ineffectual at some time points, and counterproductive at others is consistent with findings yielded in other, international universal intervention studies, that typically show no or small to moderate effects (Cuijpers, 2003; Roberts et al., 2008; Conduct Prevention Research Group, 2010). Our findings indicate, however, that primary school teachers can be trained and supported to implement universal mental health promotion programs effectively in schools, when they receive coaching and support in addition to the regular training for the programs. The current research suggests that teacher training alone is not sufficient to ensure that teachers impart mental health promotion strategies to their pupils. They also need ongoing support and coaching throughout the school year, if their students are to learn and integrate mental health strategies.

The results for suicidal ideation are similar to the findings for pro-social behaviors. Compared to the control condition, AOP implemented with both training and coaching decreased the incidence of suicidal ideation at the end of Grade 7 as students were making their transition to Secondary school. In contrast, compared to the control group, AOP implemented with training increased the incidence of suicidal ideation at the end of Grade 7—although the increase was not statistically significant. These findings suggest once again that training alone for teachers was not sufficient to protect students. This may have been because teachers did not have sufficient aid to individualize the program for children in their classes.

This is the first study to show effects for the prevention of suicidal ideation with a CBT-based universal intervention in a randomized controlled trial in Grade 7 students. The fact that prevention effects for suicidal ideation were found after the implementation of OTS is consistent with previous research, which shows that optimism is a protective factor for non-suicidal self-injury (Tanner et al., 2013). In the present study, however, optimism was not assessed and suicidal ideation rather than non-suicidal self-harm was assessed, although the latter has been shown to be a risk factor for suicidality (Skegg, 2005). Future research needs to include optimism, non-suicidal self-harm and suicidality when investigating the mechanisms of change for OTS. Nevertheless, this is the first study to suggest that the effects of optimism may generalize beyond self-harm to suicidality. Given the enormous burden of suicidality and self-harm in youth, the AOP (OTS and SLS) has the potential to reduce this burden when run universally in schools nation-wide. It should also be noted, however, that effects on suicidality were found after the parenting program was implemented, so the relative effects of the SLS, OTS and parenting program to the suicidality outcomes need to be identified in future studies.

The AOP for PF provided in Grade 7, may have helped reduce suicidal risk in the follow-up assessment at the end of Grade 8 but its impact on the mental health of the participants in the current study remains unclear and requires further investigation. The contents include guidelines and skillful strategies for parents to help their children manage challenging situations in the pre-adolescent years. Although program dose was difficult to assess, due to a limited number of returned logbooks, it appears that the PF program enhanced parents’ abilities to support their children through the social and emotional changes associated with puberty and the transition to high school. However, it is recommended that a structured parenting program consisting of workshop activities and role plays, is incorporated as part of the AOP suite in order to optimize child and parent outcomes. Research has shown that parenting programs can be one of the best strategies for preventing mental health issues in children (Bunting, 2004). Wasik et al. (2013) recommend an additional home visiting program, e.g., once a week for a few months to increase the intervention dosage and thereby increasing the probability of successful program implementation.

Our findings suggest that increases in pro-social behaviors and decreases in the incidence of suicidal ideation in a student population are linked to teacher training and support. School psychologists, counselors or specialized curriculum teachers were trained to provide this support. Coaching activities that supported the AOP trained teachers included assistance with individualizing the program to meet the needs of their students, advice on how to implement certain activities and motivate students, advice on how to adapt the content for children with special needs, encouraging parent participation, and assistance in dealing with referrals for children with serious problems. An important direction for future research is to determine the active ingredients of this coaching support and the most effective level or dose for coaching sessions. It is clear that training alone in program implementation is not sufficient to affect significant mental health outcomes for students. Previous studies indicated that program content, intensity, frequency, duration, and threshold of the training dosage are crucial factors for program effectiveness (Zaslow et al., 2010; Wasik et al., 2013).

## Limitations and Strengths of the Current Study

Some methodological issues need to be considered when interpreting the results as these may have affected the research outcomes. First, contrary to expectations, no effects were found for internalizing problems or disorders, with effects being limited to pro-social behaviors and suicidality. One possible reason for this is that the overall incidence of anxiety and depression in this age group for a universal sample is relatively low (Roberts et al., 2008). In addition, more sensitive screening measures for internalising problems, such as the Child Depression Inventory (CDI, Kovacs, 1985) and the Spence Child Anxiety Scale (SCAS, Spence, 1998) could have been included, which may have increased the likelihood of finding significant effects for anxiety and depression outcomes in this sample. Cuijpers (2003) recommends increasing the sample to approximately 30,211 subjects in both, experimental and control groups, in order to see significant reductions in the experimental group. Thus, this study was limited to the investigation of a small number of internalizing disorders. While internalizing disorders begin to increase in adolescence, they are still relatively rare in the first year of High School. This, together with the loss of a number of students at follow-up may have led to the limited effects for incidence and recovery from clinical diagnoses at follow-up.

Second, this study was further limited by the lack of measurement of externalizing problems and comorbid disorders. In addition, internalizing symptoms decreased in all groups over the period of the study. This might reflect the fact that, at all assessment points, the parents of any student identified as being at risk or with a diagnosis of anxiety or depression were confidentially notified, so that they could seek professional help outside of the school context. As such, screening for mental health conditions and informing parents of concerning results appears to be an intervention in itself, and seems to reduce the likelihood of finding differences between the intervention and control groups. Future studies should examine potential mediators of change with regard to internalizing symptoms to more fully understand the impact of the program on anxiety and depressive symptoms and disorders. Third, as the intervention was conducted in natural settings, there is a possibility that the treatment—related improvements may be influenced by teacher allegiance effects. Teachers’ fidelity, commitment and enthusiasm to implement the programs increase the likelihood of successful implementation (Pierangelo and Giuliani, 2008). It is recommended that this aspect be examined OR is examined and taken into consideration when evaluating any new programs implemented at school.

The strengths of this study indicate that universal prevention programs can impact on child and adolescent mental health. Efficacy was found for both the universal and indicated samples. Hence, the AOP could be used for population inoculation or for indicated groups in schools (Mrazek and Haggerty, 1994).

This study found that mental health programs in schools can be implemented with relative fidelity by regular school teachers with only 2 days of teacher training and a small amount of ongoing coaching and support. This finding bodes well for the sustainability of programs like Aussie Optimism and mental health promotion programs generally.

## Conclusion

This research investigated the efficacy of an enhanced version of the AOP. The design incorporated a large sample size and used a cluster randomized control trial, to take into account the clustering of students in schools. It involved a large number of schools, teachers and students. Therefore, the results have significant credibility. Aussie Optimism with teacher training plus coaching resulted in the best outcomes, namely, lower levels of suicidality and higher levels of pro-social behavior at various post-test assessments.

## Ethics Statement

This study was carried out in accordance with the recommendations of the Curtin Research Ethics Committee with written informed consent from all subjects. All subjects gave written informed consent in accordance with the Declaration of Helsinki. The protocol was approved by the Curtin Human Research Ethics Committee.

## Author Contributions

CR revised the draft and was PI on the grant that financed the study. RK contributed to methodology and data analysis. RR and NB contributed to “Introduction” and “Discussion” sections of the manuscript. YP is the coordinator of the project and contributed to conceptualization of the project. SH revised the final draft and contributed to the “Discussion” section of the manuscript. DC, SZ, and SS revised the draft and conceptual input, and were CI is on the grant that financed the study. MC edited the draft of the manuscript.

## Conflict of Interest Statement

The authors declare that the research was conducted in the absence of any commercial or financial relationships that could be construed as a potential conflict of interest.

## Footnotes

1 School SES was determined using the “H-Index,” developed by the Department of Education to assess the level of disadvantage in schools in Western Australia. Values range from 1 to 10, with 10 being the most disadvantaged and 1 the least (Department of Education, 2003).
